# Semi-Quantitative Risk Assessment of African Swine Fever Virus Introduction in Outdoor Pig Farms

**DOI:** 10.3390/pathogens12050709

**Published:** 2023-05-12

**Authors:** Alessia Rusinà, Francesco Valentini, Annalisa Scollo, Giorgio Franceschini, Sara Salvato, Veronica Cappa, Alessandro Bellato, Alessandro Mannelli, Silvia Bellini

**Affiliations:** 1 Department of Veterinary Sciences, University of Torino, 10095 Grugliasco, Italy; alessia.rusina@gmail.com (A.R.); francesco.valentini@hotmail.com (F.V.); annalisa.scollo@unito.it (A.S.); giorgio.franceschini1991@gmail.com (G.F.); alessandro.bellato@unito.it (A.B.); alessandro.mannelli@unito.it (A.M.); 2 Sorveglianza Epidemiologica, Istituto Zooprofilattico della Lombardia ed Emilia-Romagna, 25124 Brescia, Italy; sara.salvato@izsler.it (S.S.); veronica.cappa@izsler.it (V.C.)

**Keywords:** African swine fever, semi-quantitative risk assessment, outdoor pig farms, biosecurity, failure mode and effect analysis

## Abstract

In a previous study, a semi-quantitative risk assessment was developed to rank pig holdings in terms of likelihood of introducing African swine fever virus (ASFV) by assessing their compliance with biosecurity and exposure to geographical risk factors. The method was initially developed for confined pig holdings, but given that ASF is endemic in wild boar of several countries, we modified the approach to make it suitable for free-range farms as well. In the current study, a total of 41 outdoor pig farms were assessed in an area where exposure to wild boar was generally high (density from 2.3 to 10.3 wild boar per Km^2^). As expected, non-compliance with biosecurity measures was frequent in outdoor farms, and the frequency of non-compliance indicated that the absence of adequate separation of pigs from the external environment was the major weakness in the farms assessed. In 46.3% of them, there was no fence or, if present, it was not adequate to avoid contact with wild boar. However, the approach adopted proved to be suitable for identifying intervention priorities to mitigate the risk of ASFV spread in free-range pig herds and for identifying the weaknesses of individual farms, as recommended by EFSA in 2021, which suggests implementing tools to improve biosecurity by favoring higher-risk farms.

## 1. Introduction

African swine fever (ASF) is a severe disease of domestic and wild pigs which is currently causing huge economic losses in the pig sector. The causative agent is a double-stranded DNA arbovirus (ASFV), which leads to almost 100% mortality of infected pigs, in the hyperacute and acute forms of infection. Subacute and chronic forms may occur, depending on the virulence of the virus. Wild boar are conspecific with domestic pigs, and they share the same susceptibility to the virus and the same clinical signs, including a high case fatality rate [1].

A major global pandemic of ASF is ongoing, and the strains which are currently circulating worldwide belong to genotype II. At present, the disease is reported in Africa, Europe, Asia and the Pacific and Hispaniola Island in the Americas. Several European Union (EU) countries are affected, and in most of them, the disease is endemic in the wild boar population. As of January 2022, ASFV genotype II was identified in mainland Italy in the wild boar of the Apennine mountains, in the northwestern regions of Piedmont and Liguria. Since then, the disease has continued to spread in wild boar, and to date, 668 cases have been reported in this area (https://www.izsplv.it/it/notizie/308-peste-suina-africana.html, accessed on 3 May 2023).

The impact and epidemiology of ASF may vary in different areas of the world and in the different pig farming systems. The disease in sub-Saharan Africa is transmitted in a sylvatic cycle among warthogs and *Ornithodoros* soft ticks [2]. For the time being, in the current global outbreak scenario, there is no evidence of tick involvement. In the current epidemiological context, ASFV is transmitted by direct contact between infected and susceptible animals, and by indirect contact with contaminated objects or feed. In the wild boar habitat, carcasses are crucial in maintaining infection in the environment, and long-lasting endemic cycles without the involvement of domestic pigs were established in the Baltic States and Poland [3].

Inadequate farm biosecurity was often associated with ASF outbreaks in domestic pigs [4]. Indeed, since there is no effective vaccine available, ASF prevention and control are based on biosecurity and the early detection of the infection. Recent studies have indicated that insufficient biosecurity and ineffective surveillance contributed significantly to the introduction and spread of the ASFV in Europe, especially in the backyard sector. The low level of biosecurity in backyard farms in Romania has facilitated the wide spread of ASFV in pigs [5]. Oļševskis et al (2016) [6] reported the results of the epidemiological investigations of ASF outbreaks on backyard farms carried out in Latvia, which reveal that the basic principles of biosecurity were not respected in these farms. Therefore, biosecurity is crucial for the control of ASF, and risk assessment for pig farms can be useful to identify critical points in biosecurity measures, before the occurrence of ASF, as targets for intervention.

Strict biosecurity measures are more difficult to implement in farms where pigs have outdoor access, which increases the likelihood of exposure to the virus circulating in other pigs or wild boar, and possibly present in the environment. In Sardinia (Italy), 40% of ASF outbreaks in domestic pigs occurred in outdoor pig farms, and the persistence of the virus was linked to illegal pig breeding in open-range conditions and pig contact with wild boar [7]. Exposure to this type of risk can also be extended to backyard pigs which, in many countries, are a high proportion of the pig population and often have outdoor access.

Outdoor pig farms are not uncommon in the EU, although the percentages vary across Member States. Currently, the EU’s strategic approach for the control of ASF provides for a general recommendation to ban the outdoor farming of pigs at least in the areas covered by Commission Implementing Regulation (EU) 2021/605 [8]. However, in some Member States, outdoor, resourced-limited farming has an important socio-economic role in rural areas. Outdoor pig farming is a traditional way of raising pigs in areas of Southern Europe, such as the Southwestern Iberian Peninsula, Mediterranean Islands and Bulgaria, where the climate is normally milder and there are conditions for keeping pigs outdoors. In general, local breeds (Black Iberian, East Balkan, Mangalica, Gascon, etc.) are used for this type of farming. In the case of Iberian pigs, outdoor husbandry plays a role in the conservation of agroforestry ecosystems and in the production of prized pork products [9]. In Italy, the Cinta Senese is bred, which is an ancient breed of pigs that do not require special care and that probably was already bred at the time of the Romans. The meat has excellent organoleptic qualities and is used for the production of products of particular value. In 1986, 81 sows and 3 boars were registered, clear data about the serious situation of extinction in which the breed was. The number of farms over the years has increased until reaching its peak in 2007 with 210 farms. The reality of Cinta Senese represents for the Italian pig sector the most important example of recovery intervention.

In recent years, a new trend has been developing in Western European countries whereby outdoor pig producers raise their pigs in either certified free-range or organic holdings. This new trend is facilitated by the increasing availability of organic feed and positive economic projections for organic livestock [10]. Organic pig farms have grown significantly in Europe, also because of increased consumer awareness of environmental concerns, and perceived high food safety level. These farms have lower productivity, but they can brand and sell pork for higher prices [11]. However, this type of farming has been linked to a higher risk of disease spreading. This is why in certain countries outdoor farming is strictly regulated by local veterinary authorities and is required to comply with biosecurity measures to avoid interaction between wild and domestic pigs [11].

EFSA, in 2021, assessed that the baseline risk of free-range pig farms for the introduction of ASFV and its spread is generally considered to be high but with high uncertainty. The application of a specific risk assessment is therefore suggested, using approaches that consider the characteristics of farming systems. Moreover, the classification of outdoor pig farms on the basis of their biosecurity level is recommended to identify priorities for intervention to reduce the risk of ASF introduction and spread [9].

Recently, in the framework of a research project, we applied a semi-quantitative risk assessment method to classify and rank pig farms in terms of the risk of introduction of ASFV, which takes into account the relative importance of the different transmission pathways [12]. The study was focused on commercial farms, but considering the current epidemiological situation and the growing relevance of the outdoor farming system, the study was expanded to outdoor pig farms.

In the present study, the method was modified to consider the characteristics of the free-range pig-rearing system and was applied in Tuscany, a region in central Italy bordering the ASF-affected area of northwestern Italy. Tuscany is home to the Cinta Senese breed of pigs, mainly farmed outdoors, and it is also characterized by one of the most abundant wild boar populations among Italian regions [13].

## 2. Materials and Methods

### 2.1. Study Area 

**Figure 1:**
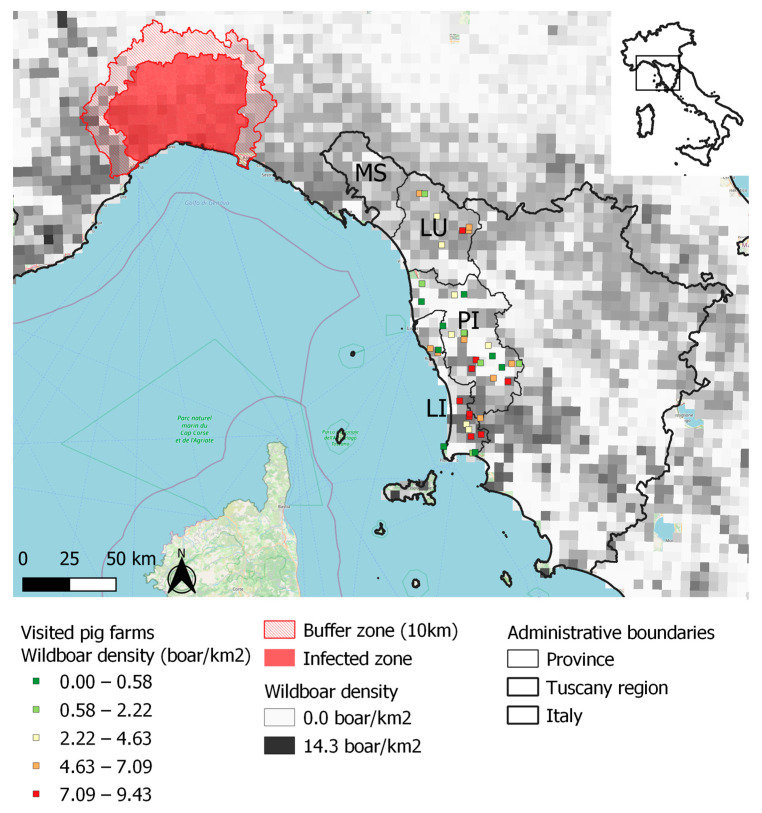
Geographic distribution of visited 41 pig farms and of the population density of wild boar in northwestern Tuscany. Farm location color is based on the Jenks natural breaks classification method. The ASF-infected area is shown. MS, province of Massa Carrara; LU, province of Lucca; PI, province of Pisa; and LI, province of Livorno, are part of the northwestern Tuscany local health unit.

The study was carried out in Tuscany. The territory comprises a wide range of natural environments, from the Northern Apennines, which is the same mountain range where ASFV has been spreading since January 2022 [14], to the Tyrrhenian coast (Figure 1). Uninterrupted wide-leaf woods, except for pastures above 1700 m above sea level, characterize the Apennines. A mosaic of woods, shrubs, crop fields, and pastures covers the hills of the central and southern parts of the study area. The wild boar population is particularly abundant, with maximum levels in wooded locations [13].

Based upon information provided by the local veterinary service, pig farming is mostly composed of non-commercial farms, accounting for 83.6% (1238 out of 1480) of recorded farms. Non-commercial farms are composed of less than 5 pigs (fatteners only), for own consumption; pigs and pig products are not commercialized. Pigs are normally kept indoors but can have outdoor access. Out of the 223 commercial farms, 121 (54.3%) are indoor, whereas the others (n = 102) are classified as “semibrado”, which are partially confined with outdoor access. These can be classified among outdoor pig farms where pigs are kept in fenced areas, with access to inside facilities [9]. Nine of the recorded farms contain wild boar for hunting purposes, seven are classified as zoos, and three are classified as other type.

### 2.2. Sample Selection

Pig farms included in the present study were selected in collaboration with official veterinarians, who carried out visits to pig farms together with a researcher to assess biosecurity. In the selection of farms, there was a specific interest in assessing outdoor farms. The sample included 41 pig farms, 35 outdoor commercial farms, and 6 outdoor non-commercial farms. Of these, 20 were in the province of Pisa (16 commercial, 4 non-commercial), 14 were in the province of Livorno (12 commercial, 2 non-commercial), and 7 were in the province of Lucca (all commercial).

### 2.3. Checklist and Data Collection

For data collection on farms, we used a checklist that was developed by Scollo et al. [12] and contained 98 items, mostly with dichotomous answers. Eighteen new items were added to the original checklist to consider specific characteristics of outdoor farming. New items included information on the following: farm structure, especially separation of the farming areas from the external environment and prevention of contact with pigs from other farms and wild boar; surveillance and data collection on the health status of animals, as critical for early detection; hygiene during husbandry practices; measures to prevent the introduction of the virus by feed and other materials. A complete list of items is shown in Table S1, supplementary material.

Items were divided into 28 so-called sub-criteria, which were assigned five-level non-compliance scores, ranging from 1 (high compliance with biosecurity measures) to 5 (lowest level of compliance), based upon the proportion of items that were assigned a positive answer, indicating compliance with biosecurity [12] (Table 1). In the few cases where sub-criteria include five mutually exclusive options, presented in increasing order of non-compliance level (e.g., B2 and B3, see supplementary material), only one of these options was chosen, based upon objective data collection in the farm: the corresponding order, from 1 to 5, was assigned as the non-compliance score for the specific item.

Sub-criteria were assigned five-level importance scores by expert elicitation, with the collaboration of eight experts from countries affected by ASF, with experience in pig management and ASF control. The importance of the original 24 sub-criteria from [12] within the six main criteria was calculated using a modified Borda method [15]. During the evaluation of the sub-criteria by the expert panel, some of the items were considered of crucial importance for biosecurity against the introduction of ASFV in pig farms, and those were defined as critical items (Table S1 in supplementary material). If one of the critical items was not satisfied, the corresponding sub-criterion was assigned the maximum non-compliance score of 5, regardless of the answer to the other items belonging to the same sub-criterion.

Conversely, the newly added four sub-criteria (within the seventh main criterion) specific to outdoor farming were all assigned maximum importance (importance score = 5), since all of them were associated with relevant risk factors for the introduction of ASFV into outdoor farming.

### 2.4. Calculation of the Risk Priority Codes by Failure Modes and Effect Analysis

The importance and non-compliance scores of the sub-criteria were used to calculate a risk priority code (RPC) for each of the seven main criteria for each pig farm, using modified failure modes and effect analysis (FMEA), as shown in the following equation:

(1) $$RPC\left(a_{i}\right)={Max}_{j}\left\{Min\left[\left(Ig_{j}\right),g_{j}\left(a_{i}\right)\right]\right\}$$

where RPC(a_i_) is the risk priority code for the criterion ai (with i = 1,…, 6); g_j_ (a_i_) is the non-compliance score for each sub-criterion j (with j = 1,…, n) included in the criterion ai (calculated as in Table 1); I(g_j_) is the importance score of each sub-criterion g_j_, included in criterion a_i_, as estimated using the Borda method; and Max_j_ is the maximum of the minimum (Min) between the non-compliance score for sub-criterion g_j_ (resulting from the checklist’s results) and the importance score that was assigned to sub-criterion g_j_.

This equation corresponds to the second analysis model described by Franceschini et al. [15], and the application to ASF risk assessment is further described by Scollo et al. [12]. The aim is to assign a high RPC for a given criterion (a_i_) to those farms that had the highest non-compliance score (corresponding to low biosecurity) on the most important sub-criteria.

### 2.5. Geographical Risk Factors

Geographic risk factors for the transmission of ASFV to susceptible farms were previously identified, and these include domestic pig density, especially if kept in farms with inefficient biosecurity, and wild boar density. In our study, we incorporated wild boar density information, obtained from a 5 km resolution raster map of predicted wild boar densities across most of Eurasia [13], into our semi-quantitative risk assessment. We imported it into R software (raster function, raster package), and predicted wild boar densities, corresponding to the locations of examined pig farms, were obtained using the extract function. Subsequently, farms were classified by Jenks natural breaks of the estimate to obtain five ordinal levels of increasing risk of exposure to wild boar, for consistency with the five levels of RPC.

The risk of transmission of ASFV between domestic pigs is a function of the distance between farms and can be modeled by transmission kernels [16]. To classify farms also in terms of local pig farm clustering, we calculated a modified G statistic [17] as an index of local spatial clustering as shown in the following equation:

(2) $$G_{i}=\sum_{j}w_{ij}x_{j}/\sum_{j}x_{j}$$

where G_i_ is an index of local clustering of pig farms around the visited farm i, xj is the number of other pig farms j, and w_ij_ is a distance kernel [16,18]. In this study, we considered local farm density, including non-commercial pig farms, in the calculation of G_i_, whereas Scollo et al. [12], who mostly focused on the main pig-producing areas in Italy, considered the density of pigs in commercial farms. In fact, the area of this study was characterized by a majority of non-commercial farms, with <5 pigs, and we preferred to use farm density as an ASF-risk indicator, rather than the density of pigs. Farms were subsequently classified by Jenks natural breaks of Gi to obtain five ordinal levels of increasing risk of local clustering of pig farms. Spatial analysis was performed using R software, version 4.1.2 (R Core Team, R Foundation for Statistical Computing, Vienna, AT, https://www.R-project.org, accessed on 4 April 2023), whereas geographic representation and Jenks natural breaks of the estimates were obtained using QGIS 3.16.2 Hannover Edition (QGIS Geographic Information System Development Team, Open Source Geospatial Foundation, http://qgis.org, accessed on 4 April 2023).

### 2.6. Land Cover Information

The raster map and polygon shapefile map of Corine land cover were downloaded from Copernicus Global Land Service (CGLS) [19]. Land use at the locations of examined pig farms was obtained from the raster map using the extract function in R software. Such information was used for descriptive purposes, and it was not incorporated into the risk score system.

### 2.7. Overall Risk Ranking of Pig Farms

Each examined pig farm was attributed ordered scores (from 1 to 5) for a total of nine indicators: seven RPCs for criteria, which were calculated from the on-farm checklist assessments, and two geographical risk indicators, corresponding to wild boar population density and local clustering of domestic pig farms. To obtain an aggregated risk index, we calculated, for each farm, the counts of decreasing values of those nine risk indicators, from counts of 5 to counts of 1. Subsequently, a risk rank was assigned to each farm by sorting them in decreasing order. This way, the highest risk was attributed to the farms that were characterized by the greatest frequency of RPCs = 5. Then, among farms with the same number of 5’s, the one with the greatest number of 4’s was classified as the highest risk. Then, the counts of 3’s and 2’s were considered. An overall ranking of farms, ordered from the farm at the highest risk of ASFV introduction (rank = 1) to the lowest, risk was obtained. Non-parametric correlation of risk rank and herd size was estimated by Kendall’s τ, using the Kendall TauB function of the *DescTools* package in R software. Kendall’s τ value is appropriate for estimating the correlation between ordinal variables in the presence of ties.

## 3. Results

**Figure 2:**
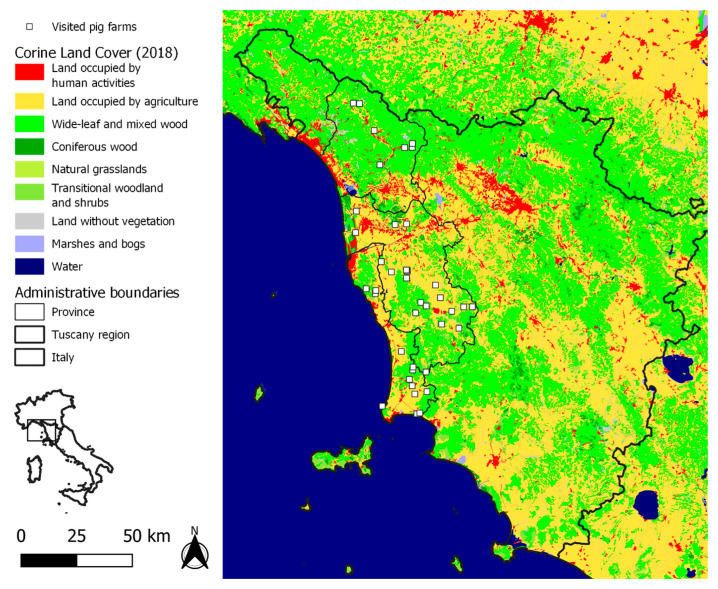
Location and vegetation cover, of 41 outdoor pig farms, visited in 2022, in northwestern Tuscany.

The majority of the 41 visited outdoor farms were at locations characterized by woodland vegetation (Figure 2), especially those in the province of Lucca, in the Apennine area, and in the southern part of the province of Livorno. Cropland was most common for farms in the province of Pisa. Few farms were in areas with shrubs or herbaceous vegetation.

Thirty-three farms contained only pigs, whereas wild boar were kept in six farms, and two had both pigs and wild boar. Twenty-six farms were classified as non-specialized, meaning that all production phases were on the same farm, from breeding to fattening. Two were breeding farms, and ten were fattening, whereas pigs in three farms were not for human consumption. The number of pigs on a farm ranged from 1 to 183; the median (first, third quartile) was 11 (4.8, 26.3).

Based upon on-farm data collection via the specific checklist, proper biosecurity measures during entrance into the farming areas by personnel and visitors were almost never implemented. Indeed, there was no clear separation from the outside, which could be identified when people access the farming area, and a Danish entry (i.e., a bench or other physical barrier that totally separates the dirty from the clean areas) was never in place. Changing or disinfecting clothing and footwear was performed in approximately 40% of the visited farms (sub-criterion A1, Table S1 in supplementary material). Employed personnel were usually absent, and this was associated with a reduced risk of ASFV introduction. Only in 4.9% of farms, owners and personnel had contact with other pig farms, whereas 26.8% of farmers were engaged in wild boar hunting. In case contact with animals from other farms or with suids (wild boar) occurred, no waiting time was observed before entering the farm (sub-criterion A2). In most farms, personnel did not bring in their own food (A3). Personnel did not take part in any training on biosecurity and disease prevention (A4). Proper recording of pigs and of pig movements from and to the farms, or within farms, was not in place in approximately 30% of the farms, whereas diagnostic tests on breeding boars were rarely performed (B1). In 29.3% of the farms, swill feeding was allowed. The introduction of pigs from other farms was rare (B2), but when introduction occurred, quarantine management was generally poor (C1). There was no particular attention to animals with impaired growth in 97.6% of farms (B3). Cleaning and disinfection on the farm were unsatisfactory in most of the farms visited (C2). Vaccination and therapy administration equipment, when present, did not undergo regular cleaning, and the recording of treatments and other production and reproduction data was incomplete (C3). Within the farms, structures were mostly inadequate to limit contact among pigs of different groups and between pigs and other animals, and structure characteristics did not allow proper cleaning and disinfection (C4). Dead pig management was generally poor, and in 95% of farms, carcasses were not properly stored (C5). Practices associated with vehicles for the transport of pigs or materials were particularly severe weak points. In fact, vehicles were never cleaned and disinfected. Moreover, practices associated with the loading and unloading of pigs were often unsatisfactory (D1–D4, E3, E4). Similarly, hygiene in feed and materials management was poor, especially regarding the clothes and footwear of operators, which were never changed when entering the farms (E1). No measure was adopted to treat materials before introduction into the farm (E2). Separation of premises from the external environment was most often incomplete, and in 73.2% of visited farms, there was no external fence for the entire farm perimeter, to prevent the entrance of wild animals and visitors (F1). Gates delimiting entrance to the farming area were absent in 48.8% of farms. Pets and livestock other than pigs were present in more than 50% of the farms, whereas disinfectants against ASFV and cleaning/disinfection protocols were absent in more than 80% of the farms (F2). Pest control was almost never implemented (F3). Structures for visitors’ hygiene, such as a dedicated parking area and a filter area, were absent in the majority of farms, and visitors were often not recorded (F4). In 78.6% of farms, no farm veterinarian would be called in case of clinical signs in pigs or reduction in reproductive and productive parameters, and in 83.9% of farms, such parameters were not recorded (G1). In 25.6% of farms, active search for dead or sick pigs, across the entire grazing areas, was not carried out on a daily basis (G1). Temporary shelter for pigs (in case of epidemics or cold season) was not available in 68.4% of farms. Feed and drink areas were not regularly controlled, and feed not consumed by pigs was not removed. The external fence was not checked at least weekly in 20% of the farms (G2). Double fences, which are a major measure to reduce the risk of direct contact between pigs and wild boar, were present in only three out of the 41 visited farms (7.3% compliance, sub-criterion G3). A “boar-proof” fence (single solid or double fence at least 1.5 m high and properly fixed to the ground to prevent the ingress of wild boar under the fence) was absent in 46.3% of the farms (G4). Feed, including straw, was not subject to preliminary storage before administration to pigs; 45.5% of farmers administered grass from out-of-farm areas, where wild boar may be present, with no previous storage or treatment (G4). Materials that were in contact with pigs were not periodically cleaned and disinfected.

### 3.1. Geographic Risk Factors 

Wild boar population density ranged from 2.3 to 10.3 heads per square kilometer, and it was highest at farm locations in the Apennine area in the province of Lucca and in the southern parts of the provinces of Pisa and Livorno (Figure 1).

**Figure 3:**
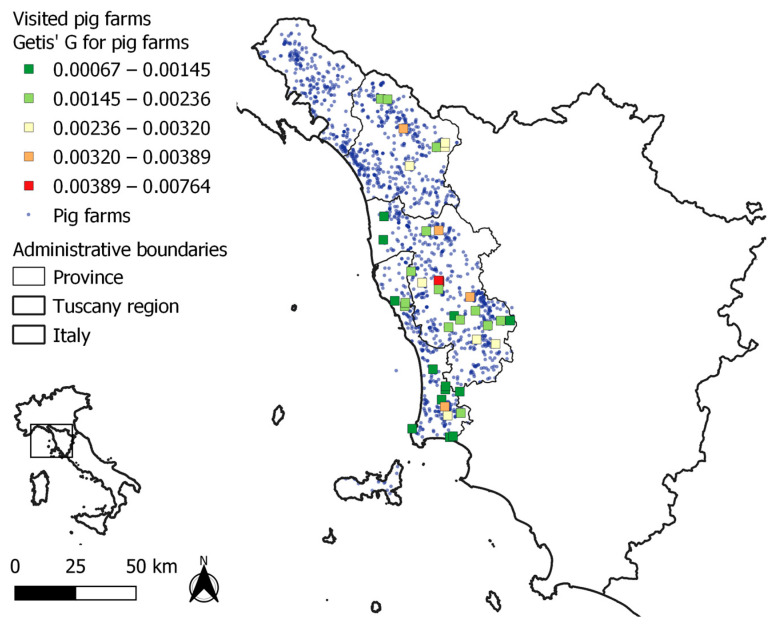
Geographic distribution of 41 visited outdoor pig farms in northwestern Tuscany, which were classified by G statistics, as an index of local clustering of pig farms. Visited farms are identified by quadrats.

The median (first, third quartile) number of farms (including non-commercial farms) surrounding each farm, within a 3 km distance radius, was 8 (3, 11). The number of pigs within a 3 km distance was 53 (5, 99) (it was calculated by including commercial farms only, since the number of pigs within non-commercial farms was not considered as accurate). The clustering of pig farms, including non-commercial farms, was highest in areas of the province of Pisa (Figure 3).

### 3.2. Risk Ranking of Visited Farms

**Figure 4:**
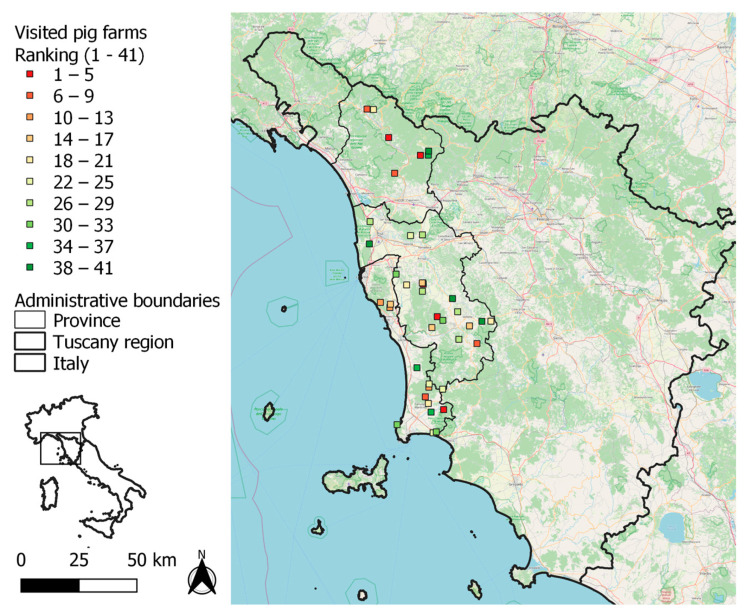
Geographic locations of 41 visited outdoor pig farms in northwestern Tuscany, classified by a modified failure modes and effect analysis (FMEA) including on-farm biosecurity compliance, as well as geographic risk factors. Low ranking corresponds to an increased risk of African swine fever virus (ASFV) introduction.

Based on our semi-quantitative approach, the highest risk of ASFV introduction was found for pig farms in the province of Lucca, and other high-risk farms were irregularly distributed across the study area, as shown in Figure 4. No correlation was detected between risk ranking and herd size by Kendall’s τ (*p* = 0.70).

## 4. Discussion

Interactions between domestic pigs and wild boar may facilitate the spread and maintenance of a range of pig pathogens, including ASFV, and contact between wild boar and domestic pigs is considered more likely to occur in outdoor production settings than in total confinement farms. This is the reason why, at the EU level, there is a recommendation to prohibit outdoor pig farming, at least in the areas affected by ASF. However, in some European Member States, this type of farming is an important socio-economic asset in rural areas, and in certain countries, autochthonous breeds of pigs are reared outdoors. A specific method to assess the risk of ASFV introduction in outdoor pig farms is recommended, with the aim to promote continuous improvement of biosecurity practices giving priority to high-risk-ranking farms [9]. Furthermore, EFSA, in 2021, considered that the risk of free-range pig farms for the spread of ASFV is generally high, and the application of risk assessment, using approaches that consider the characteristics of outdoor farming systems, is recommended.

In this light, in the present study, we integrated an ASF risk assessment method developed within the framework of the project DEFEND to also consider the characteristics of the outdoor pig farming system. In our semi-quantitative method, we considered the ordinal property of scores of non-compliance to biosecurity and geographic risk factors, and of importance scores. In addition to the identification of weak points in biosecurity, the final result of our approach was a ranking of pig farms according to their risk, to be used in the prioritization of intervention to prevent ASF. In our study, the herds at greatest risk were identified in the Apennine areas of northwestern Tuscany (Figure 4) as the result of high RPCs of biosecurity criteria. A high population density of wild boars contributed to relatively great risk both in the province of Lucca and in the southern parts of Pisa and Livorno. ASF is currently present in wild boars in northwestern Italy (Figure 1), and their movements along a continuous wood cover might lead to the spread of the infection to the Apennines of our study area (Figure 2). In our study area, the densities of farms and of farmed pigs are low in comparison with those in the main Italian pig-production regions. Nevertheless, the combination of traditional husbandry systems (outdoor and non-commercial farms) and wild boar population density may favor the spread and maintenance of ASFV, following its introduction. Geographical variations in Italy agree with findings from other European countries, where the risk of ASF is affected by the characteristics of domestic pig production (in particular, the proportion of so-called backyard holdings), geographic factors (including topography, natural barriers), and the characteristics of the wild boar population [3,4,5,20,21]. In Slovakia, as an example, the PCR-positive results for ASF in domestic pigs were highly correlated with the density of small-size farms (fewer than 10 animals) per district [3], and the probability of ASF-positive wild boar was strongly associated with the density of pigs in small holdings [4,20,21,22]. The weak points in biosecurity, identified in outdoor pig farms, were similar to the ones previously detected in Europe, and in a limited sample of non-commercial and small commercial pig farms in Italy [12]. Indeed, the absence of adequate separation of pigs from the external environment was a major weakness observed in the farms assessed in this study. The lack of adequate fences separating outdoor pig farms from the external environment (G3, Supplementary Material Table S1) is a major critical point in preventing ASFV introduction, especially in areas where wild boar or other pigs raised outdoors are present. Accordingly, in a previous study, experts were 66–90% certain that proper external fences would reduce the baseline risk of ASF in outdoor pig farms by more than 50% [9]. It is worth mentioning that adequate structures may require a considerable financial investment when farms extend over large areas. On the other hand, weak points at the farm-structure level, such as the lack of barns to confine pigs during epidemics, could threaten the conservation of certain native pig breeds for which the production regulations provide for outdoor rearing.

One of the important findings of the study is that relating to the feeding of swill to pigs, which is subject to a total ban in the EU (EC Regulation, 2009), given that it is the most likely route of transmission of serious diseases of pigs, including ASF. Despite that, it was still practiced in outdoor pig farms in 29.3% of the farms visited. Furthermore, as expected, it was found that cleaning and disinfection of the facilities, transport vehicles, and equipment and personnel hygiene are difficult to apply in outdoor pig farms. However, considering the characteristics of resistance of ASFV [1], the presence of a protocol for the implementation of these activities is part of the reinforced biosecurity considered in the EU legislation [8]. Therefore, training of farm personnel on biosecurity measures, which was previously shown as insufficient by Scollo et al. [12], should also include information on the risk associated with outdoor farms [23].

When we integrated the original biosecurity checklist [12], with the additional items relevant to free-range pig farming, we included also the evaluation of the procedure implemented to monitor the health status of animals, which might be difficult to apply in outdoor farms (G1, Supplementary Material Table S1). The non-compliance with this sub-criterion may hamper the early detection of the disease in outdoor farming, which, together with the delayed removal of dead pigs, may increase the likelihood of ASFV spread. The evaluation of biosecurity measures to reduce contact among animals inside the farm or with the out-of-farm environment (G2, Supplementary Material Table S1) indicated other important weak aspects to consider. Indeed, regular checking of feed and drink sites and removal of feed debris, as an example, if not implemented, attract wild animals and therefore may represent a risk in ASF-affected territories. Even manure, if not properly managed, could be a source of environmental contamination by infectious agents. Access by visitors, including tourists, increases the risk of ASFV introduction, and regular checks of external fences might be difficult in large, outdoor farms [9].

In the new sub-criterion G4 (Supplementary Material Table S1), we considered the administration of grass or other fresh fodders to pigs, as well as the introduction of straw as litter, which is relatively frequent in outdoor farms. Therefore, the risk of ASFV introduction via feed and other materials without treatments to inactivate the virus could be relevant.

Priority to improve biosecurity should be given to farms characterized by high RPCs and high levels of exposure to geographical risk factors. However, to successfully improve biosecurity, interventions must be feasible and sustainable in time [9,12]. Biosecurity criteria are often under the direct responsibility of farmers, and they are influenced by the farm manager’s decisions and investments. In contrast, geographic risk factors, such as the local density of pig farms and wild boar population density, are beyond the control of individual farmers and should be the object of national or regional disease prevention programs.

The calculation of RPCs by FMEA allows for the integration of biosecurity measures of different types. Such flexibility makes it suitable also for outdoor pig farming in different geographic areas since it can be adapted to variable husbandry and environmental conditions. It is possible to add checklist items and sub-criteria without affecting the validity of the method. Moreover, the transparency of all stages of the risk assessment process and of the calculation of the RPC, which allows a transparent assessment both at the farm level and in a certain territory, must be seen as a useful tool for the competent veterinary authorities for a critical evaluation of the situation and to direct control measures and resources towards the most influential hazards and the most relevant production system for the specific geographical area.

In the current epidemiological context, it should be considered that ASF is endemic in wild boar in several EU countries and that the disease is spreading in the wild, also by territorial contiguity. Based on these circumstances, one wonders how sustainable it is, in the long term, to ban outdoor pig farming per se, given that in some areas this type of farming has an important socio-economic role and that in certain instances it was also encouraged and supported with EU funds to promote alternative ways of farming or to sustain endangered breeds.

## Figures and Tables

**Table 1:** Description of the sub-criterion non-compliance scoring system.

Sub-Criterion Non-Compliance Score	Description
1	All items are satisfied
2	Between 62.6% and 99.9% of the items are satisfied
3	Between 37.6% and 62.5% of the items are satisfied
4	Between 0.1% and 37.5% of the items are satisfied
5	No items are satisfied, or at least one “critical item” is not satisfied

## Data Availability

Inquiries regarding data supporting reported results can be directed to the corresponding author.

## Supplementary Materials

The following supporting information can be downloaded at: https://www.mdpi.com/article/10.3390/pathogens12050709/s1, Table S1: Complete list of the 116 items considered in the checklist and non-compliance (negative answer to dichotomous answers) in 41 outdoor pig farms, in northwestern Tuscany.
