# Supplementary material for: Semi-Quantitative Risk Assessment of African Swine Fever Virus Introduction in Outdoor Pig Farms
Source: Pathogens. 2023 May 12;12(5):709. doi: 10.3390/pathogens12050709 (PMC10222819; doi:10.3390/pathogens12050709)
Supplement: Supplementary file 1 [file pathogens-12-00709-s001.zip › pathogens-2357789-supplementary.pdf]

Supplementary Material

Table S1 Complete list of the 116 items considered in the checklist, and non-compliance (negative answer to dichotomous answers) in 41 outdoor pig farms, in northwestern Tuscany.
*items that required the selection of one among 5 mutually exclusive options, of increasing non-compliance level (from 1 to 5) with optimal biosecurity practices.

Criterion	Item id	Sub-criterion	Item description	% of non-compliant farms (number of farms with non-missing answers)
A: personnel	1	A1 Entrance of personnel into the farm	Change of clothes and footwear is carried out **(critical item)**	41.5 (41)
	2		Clothing and footwear are cleaned and disinfected regularly or are disposable.	41.5 (41)
	3		There is a locker room with toilets and cleaners and a clear separation between clean and dirty areas: the entrance to the locker room (dirty) is DIFFERENT from the exit (clean), so that there are no areas of overlapping dirty-clean transit	97.6 (41)
	4		There is a Danish entry, i.e. a bench that totally separates the dirty and clean areas and remember the personnel the threshold	100.0 (41)
	5		Personnel take a shower before entering the farm	97.6 (41)
	6	A2 Contact of personnel with other pigs and wild boar hunting	The staff has no other pigs **(critical item)**	0.0 (41)
	7		Staff has no contact with other pig farms **(critical item)**	4.9 (41)
	8		Staff does not engage in wild boar hunting **(critical item)**	26.8 (41)
	9		If it comes into contact with animals from other farms or suids (wild boars), the staff should rest for at least 48 h before entering the farm after this activity.	100.0 (13)
	10*	A3 Food introduction by personnel	There is a ban on staff bringing in their own food, or there is an in-house canteen.	92.7 (41)
			Staff may bring in their own food, but it may only be consumed in a defined area, the area is physically separated from the animal holding area, and staff may only consume food during designated breaks	0.0
			Staff may bring in their own food, but it may only be consumed in a defined area, the area is physically separated from the animal holding area, staff may consume the food at any time during the day	2.4
			Staff may bring in their own food, but it may only be consumed in a defined area, the area is not physically separated from the animal holding area, staff may consume food at any time of the day or during established breaks	0.0

			Staff may bring in food, and there is no indication as to the area in which it must or may be consumed	4.9
	11*	A4 Personnel training	All personnel working on the farm (workers, technicians, managers, keepers/owners) are adequately trained on biosecurity and the risks of intro ASF virus and the course is less than a year old (documentation)	0.0 (41)
			All personnel working on the farm (workers, technicians, managers, keepers/owners) are adequately trained on biosecurity and the risks of intro ASF virus and the course is more than a year old	0.0
			Only a portion of the staff working on the farm are trained in biosecurity and the risks of introducing ASF, and the course is less than a year old	2.4
			Only a portion of the staff working on the farm are trained in biosecurity and the risks of introducing ASF, and the course is more than a year old	2.4
			The farmer and staff are either not trained at all on biosecurity and the risks of introducing ASF or there is no clear evidence of courses	95.1
B: Animal introduction and management	12	B1 Health/feeding /breeding status of introduced pigs	The health status of all animals for ASF is known, prior to their entry into the herd	Not applicable (0)
	13		All animals are correctly identified on the farm	37.5 (40)
	14		There is an accurate and punctual registration of movements both in/out and within the holding structures	27.5 (40)
	15		The seminal material comes from authorized semen collection centers or the reassembly is carried out by in-house breeders or, in the case of natural service, the boars have undergone the appropriate diagnostic tests.	85.7 (21)
	16		Animals are not fed catering waste, canteen waste or household leftovers (swill feeding) **(critical item)**	29.3 (41)
	17		Is family slaughter, if it occurs, done under the supervision of a veterinarian? (Documentation) Only for family farms	6.7 (15)
	18*	B2 Number of farms of origin of the introduced pigs	The farm introduces animals throughout the year from one or two farms	100.0 (41)
			The farm introduces animals throughout the year from three farms	0.0
			The farm introduces animals throughout the year from four farms	0.0
			The farm introduces animals during the year from five farms	0.0
			The farm introduces animals during the year from more than five farms	0.0

	19*	B3 Management of animals with an impaired growth	There are no "scraps" (animals with impaired growth) they are all euthanized and the vehicle collects the scraps from a single collection point	97.6 (41)
			Scraps are taken away at the end of the cycle and the vehicle collects them from a single collection point	0.0
			Scraps are taken away at the end of the cycle and the vehicle does not collect it from a single collection point	0.0
			Scraps are taken away during the cycle and the vehicle collects them from a single collection point	2.4
			Scraps are taken away during the cycle and the vehicle does not collect them from a single collection point	0.0
C: Animal shelters management	20	C1 Quarantine	A quarantine of at least 30 days is carried out (with animals serologically tested and visited)	83.3 (30)
	21		There is a physically separate dedicated premise for quarantine	60.0 (25)
	22		Biosecurity measures for staff access to the quarantine area are implemented	87.5 (8)
	23		Quarantine premises have separate entrances	22.2 (9)
	24		Quarantine premises have separate pits	100.0 (2)
	25		All-in/all-out and a suitable sanitary break period is practiced for the quarantine premises	50.0 (8)
	26		For quarantine management, staff change clothes entirely and use dedicated equipment	62.5 (8)
	27	C2 Internal animal flow and cleaning and disinfection procedures	Management of all-in/all-out: it is complete, no temporal overlapping of batches in the whole farm or quarantine is carried out	63.9 (36)
	28		At least 5 days of sanitary break between emptying and introducing the new animals is followed	66.7 (30)
	29		Structures are meticulously washed using disinfectants between batches	85.0 (20)
	30		Pressure equipment is available for cleaning, washing and disinfection	37.5 (40)
	31	C3 Vaccine prophylaxis and treatments for other infectious diseases	There is a hygienic and sanitary procedure/protocol of handling equipment used for vaccine prophylaxis and therapeutic treatments	100.0 (8)
	32		Any results of official or self-inspection analyses are present (samples taken from animals or other matrices)	33.3 (36)
	33		There is a documented vaccination plan	27.8 (36)
	34		There is a system for recording company health, breeding, reproduction and production data	62.2 (37)

	35	C4 Structure and buildings	The farm has physical or natural barriers that circumscribe the animal housing and management area	14.6 (41)
	36		The area of housing and management of animals, has a wall or a fence suitable to prevent the entry of other animals including wild ones	75.6 (41)
	37		There is a planimetry, with sheds and stalls univocally numbered, through which it is possible to verify the unidirectional flow of the movements of the animals in the farm and identify the groups of animals.	70.6 (34)
	38		Animals do not have the ability to access/live in the outdoor environment, or animals do have the ability to access only an external dunging area totally confined by a solid wall 1.5 m high.	85.4 (41)
	39		Livestock housing shall have airtight, well-maintained, seamless walls, floors, and windows and doors that can be effectively cleaned and disinfected	52.9 (17)
	40	C5 Dead pigs' management	Where piglet carcasses are temporarily stored on the farm premises, pending their removal, the containers used shall be properly sealed and suitable for storage	73.2 (41)
	41		Dead animal carcasses shall be removed from farm premises within 24 hours of death.	26.8 (41)
	42		Dead animal carcasses shall be stored in a suitable, functioning insulated container or in a sealed cold storage room for disposal in accordance with health regulations	95.0 (20)
	43		The area underneath the carcass container/cold storage room, is suitable both for the collection of any leaking materials or liquids and for cleaning and disinfection	0.0 (1)
	44		The area underneath of the carcass container/cold storage room, is suitable for cleaning and disinfection	0.0 (1)
D: Animal transport vehicles	45	D1 Live animal transport vehicles	There is an area for the disinfection of live animal transport vehicles located near the access to the farm but separate from the area designated for housing.	87.8 (41)
	46		No live animal transport vehicles enter the farm's clean area	68.4 (38)
	47		All farm vehicles do not have access to other pig farms	50.0 (40)
	48		Vehicles are cleaned and disinfected (including wheels and fenders) with disinfectants proven effective for ASF before entering the clean area	100.0 (37)
	49	D2 Live animal unloading/loading	There is a loading bay for loading and unloading animals	94.6 (37)
	50		Specific gates are in place to prevent animals from turning back	96.0 (25)

	51		The unloading/loading of live animals takes place outside the farm's clean area	72.7 (33)
	52		The unloading/loading of live animals takes place with a single-load vehicle.	16.1 (31)
	53	D3 Carcasses disposal	The carcass container/cold storage rooms are outside the clean area of the farm	0.0 (1)
	54		Emptying the carcass container/storage room takes place by non-contact tipping directly into the carcass removal truck	Not applicable (0)
	55		The contents of the carcass container/cold storage rooms are transported by staff to the outside of the farm	10.0 (10)
	56		The carcass removal truck does not enter the farm	12.5 (8)
	57	D4 Equipment and tools for loading/unloading live animals	While loading animals, transporters help inside the truck but never enter any clean area of the farm, which is clearly demarcated **(critical item)**	66.7 (21)
	58		Clothing provided to transporters is company or freshly laundered, and boots are company issued **(critical item)**	23.8 (21)
	59		The loading bay and the tools used are never switched from the dirty to the clean area and vice versa	50.0 (14)
	60		The loading bay and the tools used are disinfected after each use	100.0 (19)
	61		The weighbridge for the live animal vehicles, if any, is located outside the animal housing and governing area	Not applicable (0)
E: Material management: feed and fodder, slurry, other vehicles	62	E1 Procedures for loading/unloading of feed and materials	The unloading of feed, bedding, outdoor material, etc. takes place outside the perimeter of the clean area.	62.5 (40)
	63		Vehicles and outside operators do not stop or pass through a clean area to reach silos/barn/depot	63.6 (11)
	64		If for the retrieval of the materials to be brought in, company operators must step on a common unloading area with external operators or pass through a dirty area, they use dedicated clothing and footwear	100.0 (10)
	65		For the collection of the material to be brought in, company operators use dedicated footwear and clothing	100.0 (39)
	66		The truck route is such that it crosses the operators' routes but the operators adopt procedures to remedy the problem or the truck route does not cross the operators' routes	66.7 (9)
	67		The silos and the hayloft are inside the farm perimeter but not in contact with animals and/or operator routes (e.g. the staff adopts procedures to avoid the problem such as the change of footwear and clothes if they cross the path of vehicles or external operators) or the silos and the hayloft are outside the farm perimeter	66.7 (3)

	68	E2 Feed and materials storage	The external material (bedding, arching material...) is stored for 30 days or treated with UV rays.	100.0 (40)
	69		Forage material is stored for 30 days or treated with UV light	100.0 (40)
	70		The material is stored in closed and covered premises	20.0 (40)
	71		Feed storage is protected from animals by sparrow nets/sealing/covering	20.0 (40)
	72	E3 Slurry management	The slurry tank is located outside the perimeter of the clean area	50.0 (2)
	73		The slurry tank can only be reached by a route outside the perimeter of the clean area of the farm	100.0 (2)
	74		The slurry tank is not accessible to wild animals	0.0 (2)
	75		There are entrances for slurry transport operations differentiated from those of the animal housing/management area	100.0 (2)
	76		Slurry of other origin are not spread in the fields adjacent to the farm	0.0 (41)
	77	E4 Vehicles for loading/unloading feed and materials	Vehicles other than those for loading/unloading live animals and carcasses (transport of feed, materials, owned vehicles) do not enter the farm's clean area.	82.9 (41)
	78		The vehicles are disinfected in advance on a dedicated yard and there is documentation	97.6 (41)
	79		Vehicles neither transit nor are parked anywhere on the farm	25.0 (40)
	80		Vehicles do not come into contact with the area where animals are present (clean area)	20.0 (40)
F: Buildings and farm planimetry	81	F1 Farm perimeter barriers	There is an external fence for the entire farm perimeter that prevents the entrance of wild animals and visitors **(critical item)**	73.2 (41)
	82		Access points are closed by a gate	48.8 (41)
	83		Signs prohibiting access for unauthorized persons are present and clearly visible at the entrance	75.6 (41)
	84		The farm has a dedicated area, located before the entrance barrier, for the parking of staff vehicles.	34.2 (41)
	85		The pavement around the farm is asphalted	100.0 (41)
	86		There is no unused or waste material around the farm, the farm is well maintained, there are no bushes, brushwood around the farm	22.0 (41)
	87	F2 Other animals and cleaning and disinfection procedures	No pets are present and have access outside the farm perimeter	56.1 (41)
	88		No other livestock species are farmed	53.7 (41)
	89		Disinfectants with proven efficacy against ASF are available **(critical item)**	82.9 (41)
	90		There is a procedure/protocol of cleaning and disinfection of housing facilities and	83.8 (37)

			equipment after the end of each production cycle.	
	91	F3 Pest and rodent control	Rodent and pest control are checked at least every <6 months and there is documentation	100.0 (41)
	92		Rodent control is carried out by an external specialized company	97.6 (41)
	93		Pest control is carried out by an external specialized company	100.0 (41)
	94		Sparrow nets are in place or it is otherwise ensured that birds cannot enter the premises	100.0 (41)
	95	F4 Visitors	The farm has a designated area, located before the entrance barrier, for visitors' vehicles to park	85.4 (41)
	96		The farm has a dedicated filter area used as an hygiene lock for visitors	80.5 (41)
	97		Rooms used as visitors' hygiene lock are adequately equipped with toilets and cleaners	22.2 (9)
	98		There is an up-to-date visitors' logbook indicating at least the date, first and last name of the visitor, reason for the visit and vehicle registration number	63.4 (41)
G: outdoor specific criterion	99	G1 Check of health status	If a quarantine area is present, animals are inspected at least on a daily basis, to detect clinical signs onset.	12.5 (8)
	100		The health status of animals is checked on a daily basis.	4.9 (41)
	101		There is farm-veterinarian, who is contacted within 24 h, in case of clinical sign onset or changes in production parameters.	78.6 (28)
	102		Outdoor farming areas are checked on a daily basis, to detect and remove dead pigs.	25.6 (39)
	103		A system is implemented to record production parameters: birth and mortality rates, food intake, weight of pigs at introduction, sale, slaughter.	83.9 (31)
	104		There are barns where all pigs could be temporarily confined, for health, or other reasons.	68.4 (38)
	105	G2 Contacts	Practices, favoring direct and indirect contacts among animals, such as sharing of containment tools, only one feeding and drinking site, mixing of groups are avoided.	35.9 (39)
	106		Pig feeding and drinking facilities are isolated, so as to prevent access of other animals, including birds and rodents.	17.5 (40)
	107		Pig feeding and drinking sites are regularly checked, and left feed is promptly removed.	100.0 (7)
	108		Access to pig farming areas is strictly limited to farm personnel, whereas visitors are excluded	22.0 (41)
	109		A protocol for manure disposal is in place	92.0 (25)

	110		The external fence is checked at least weekly, and always following strong wind, heavy rain, or snow, and it is correctly maintained and repaired if needed.	20.0 (40)
	111*	G3 External fence	The farming area is delimited by a double, 1.5 m high, metal fence, with 1 m of minimum distance between fences, or by a wall (solid fence), extending 20 or 30 cm underground.	7.3 (41)
			The farming area is delimited by a single metal fence, or by a wall 1.5 m high, 20 or 30 cm underground, in association with an internal electrified double wire, at 20, and 40 cm from the ground.	0.0
			The farming area is delimited by a single metal fence, or by a wall 1.5 m high, 20 or 30 cm underground. Alternatively, double fence, with only one of adequate height and underground part.	41.5
			The farming area is delimited by a single metal fence, or by a wall 1.5 m high, not extending 20 or 30 cm underground. Alternatively, double fence, not extending underground	4.9
			The farming area is delimited by a fence, which is not compliant with any of the requirements, or there is no fence.	46.3
	112	G4 Feed and other materials	Personnel washes hands before access to farming area.	100.0 (7)
	113		Pigs are not administered grass or fresh fodder, unless those are treated to inactivate ASFV, or stocked in closed rooms for 30 days before being fed to pigs.	45.5 (33)
	114		Straw is not used as litter for pigs, unless it is treated to inactivate ASFV, or stocked in closed rooms for 90 days before use.	41.4 (29)
	115		No tool, machinery, or feed is exchanged with other pig farms.	5.4 (37)
	116		Materials and tools being in contact with pigs are periodically cleaned and disinfected	100.0 (17)
